# Association between very advanced maternal age and adverse pregnancy outcomes: a cross sectional Japanese study

**DOI:** 10.1186/s12884-017-1540-0

**Published:** 2017-10-10

**Authors:** Kohei Ogawa, Kevin Y. Urayama, Shinji Tanigaki, Haruhiko Sago, Shoji Sato, Shigeru Saito, Naho Morisaki

**Affiliations:** 1 Center for Maternal-Fetal, Neonatal and Reproductive Medicine, National Center for Child Health and Development, 2-10-1 Okura, Setagaya-ku, Tokyo, 157-8535 Japan; 2 Collaborative Departments of Advanced Pediatric Medicine, Graduate School of Medicine, Tohoku University, 1-1 Seiryo-cho, Aoba-ku, Sendai-shi, Miyagi 980-8575 Japan; 3 Department of Social Medicine, National Center for Child Health and Development, 2-10-1 Okura, Setagaya-ku, Tokyo, 157-8535 Japan; 4 Graduate School of Public Health, St Luke’s International University, 9-1 Akashi-cho, Chuo-ku, Tokyo, 104-8560 Japan; 5 Perinatal Center, Oita Prefectural Hospital, 476 Bunyo, Oita-shi, Oita 870-8511 Japan; 6 Department of Obstetrics & Gynecology, University of Toyama, School of Medicine, 2630 Sugitani, Toyama-shi, Toyama, 930-0194 Japan

**Keywords:** Assisted reproductive technology, Birth outcome, Effect modification, Japanese, Pregnant women of advanced age, Parity

## Abstract

**Background:**

While several studies have demonstrated the increased risk of pregnancy complications for women of advanced age, few studies have focused on women with very advanced age (≥ 45), despite the increasing rate of pregnancy among such women. Furthermore, how such risks of increase in age differ by maternal characteristics are also poorly understood. Thus, we aimed to clarify pregnant outcomes among women with very advanced age and how the effect of age differs by method of conception and parity.

**Methods:**

We used the national multicenter Japan Society of Obstetrics and Gynecology perinatal database, including 365,417 women aged 30 years or older who delivered a singleton between 2005 and 2011. We divided women into four groups based on age (years): 30–34, 35–39, 40–44, and ≥45, and compared risk of adverse birth outcomes between the groups using Poisson regression. Effect modification by parity and use of assisted reproductive technology (ART) was also evaluated.

Results: Compared with women aged 30–34 years, women aged 45 or older had higher risk of emergency cesarean delivery [adjusted risk ratio (aRR): 1.77, 95% confidence interval (95% CI): 1.58–1.99], preeclampsia (aRR: 1.86, 95% CI: 1.43–2.42), severe preeclampsia (aRR: 2.03, 95% CI: 1.31–3.13), placenta previa (aRR: 2.17, 95% CI: 1.60–2.95), and preterm birth (aRR: 1.20, 95% CI: 1.04–1.39). The effect of older age on risk of emergency cesarean section, preeclampsia, and preterm birth were significantly greater among those who conceived naturally compared to those who conceived by ART. The effect on emergency cesarean section was stronger among primiparous women, whereas the risk of preeclampsia associated with older age was significantly greater among multiparous women.

**Conclusions:**

Very advanced maternal age (≥ 45) was related to greater risk for adverse birth outcomes compared to younger women, especially for maternal complications including cesarean section, preeclampsia, severe preeclampsia, and placenta previa. The magnitude of the influence of age also differed by conception method and by parity.

## Background

Pregnancy at advanced maternal age (over 35 years) has increased in many high income countries over the past several decades, [1–3] with recent rates reported to be as high as 9.1% in the US, [4] and 28.1% in Japan [5]. In Japan, one of the Asian countries which has experienced a considerable increase in average age at pregnancy, the number of births from women of very advanced age, such as 40–45 and ≥45 years of age, has also surged, with recent numbers in 2015 to be 52,557 (5.2%) and 1038 (0.1%), respectively [5]. Such increase in average maternal age has also been observed in many parts of Asia, such as Korea, [6] China, [7] and Taiwan, [8] which may be attributable to the increase in women’s participation in society in these countries.

A number of studies have demonstrated that pregnancy among women of advanced age is associated with increased risk of pregnancy complications and adverse perinatal outcomes, such as gestational diabetes mellitus, preeclampsia, placenta previa, cesarean section, preterm birth, low birthweight, maternal mortality, and perinatal mortality [9–13]. However, most studies have focused on adverse outcomes among women aged ≥35, or ≥40, [14] and the few which have studied birth outcomes of pregnancies of older women (i.e., over 45 years of age) suffer from limitations. For example, most studies were conducted nearly 20 years ago, [15–17] when such women were likely to be multiparous and have conceived naturally without artificial reproductive technologies (ART), unlike the women who conceive at similar ages today [18–20]. Furthermore, it is conceivable that the effect of older age on the risk of adverse birth outcomes, such as cesarean delivery and preeclampsia, may significantly differ by method of conception and parity because women who conceive by ART have a higher risk for a number of adverse perinatal outcomes, and parity also has a significant effect on the risk of cesarean section and preeclampsia [21–23]. However, only one recent study conducted in 217 women in Australia [19] considered this potential effect modification on a limited number of birth outcomes.

In order to address the yet unanswered questions related to this association, we used the Japanese national multicenter-based delivery registry, which includes a relatively large group of women of advanced age, and evaluated the association between adverse birth outcomes and very advanced maternal age, and whether this association differed by maternal characteristics, namely parity and method of conception. Such information would be useful to clinicians when providing antenatal counseling to women of very advanced age.

## Methods

### Study population

This cross sectional study was conducted based on the Japan Society of Obstetrics and Gynecology Perinatal Database (JSOG-DB), an ongoing registry based currently on 149 Japanese tertiary hospitals and covers nearly a tenth of all births in Japan, with over a hundred thousand births registered each year [24]. For this database, maternal demographics, pregnancy complications and birth outcomes were transcribed from medical charts in each hospital using a standardized format.

Multiple pregnancies are at higher risk of adverse outcomes compared to singleton pregnancy, and conception with ART is associated with both older age and multiple pregnancies. [25] Therefore, to differentiate the direct effect of maternal age on birth outcome from any indirect effect mediated by multiple pregnancies, [26] we included only women with singleton pregnancies. Similarly, we excluded women carrying a fetus with congenital abnormalities, as these women have a higher risk of adverse outcomes. Also, as the risk of adverse pregnancy outcomes in women of younger age is strongly related to social risk factors, [27] we restricted our sample to 370,964 women aged 30 years or older who gave birth to singletons with no congenital anomaly between April 2005 and December 2011. From this population, we excluded 5547 women with missing data on either gestational age (*n* = 207), birthweight (*n* = 2023), mode of delivery (*n* = 2210), and those with unreliable combination of birthweight and gestational age using the criteria proposed by Alexander et al. [28] (*n* = 1107). Among the other variables, smoking status, maternal height, pre-pregnancy body mass index (BMI) and gestational weight gain were missing in a large number of women. An additional 4393 had extreme values (> + 4SD or <−4SD) of height, BMI or gestational weight gain, thus we considered these data to be unreliable. To address these issues while maximizing our sample size to maintain the potential for a generalizable and robust analysis, we performed multiple imputation on the missing and unreliable data and pursued the main analysis on 365,417 women. These results were subsequently confirmed in a sensitivity analysis on the subset 183,084 women after excluding those with missing or unreliable data on height, BMI or gestational weight gain, and including “missing” as a smoking status (yes, no, missing).

For multiple imputation, we replaced missing or unreliable data with 30 sets of imputations for the following variables: maternal height (*n* = 157,767), maternal BMI (*n* = 120,257), maternal gestational weight gain during pregnancy (*n* = 134,122) and smoking (*n* = 153,652). For imputation, we used multivariate imputation by chained equations, which does not require the assumption of a multivariate normal distribution, and uses a series of regression models where each variable with missing data is modeled conditional upon the other variables in the data.

### Variables of interest

The primary exposure of interest was maternal age. Pregnant women were categorized into 4 categories: 30–34 years of age, 35–39 years of age, 40–44 years of age, and 45 years of age and older. Women 30–34 years of age was considered the reference group [19].

We considered a variety of adverse birth outcomes captured in our database: preterm birth, very preterm birth, extremely preterm birth, small for gestational age (SGA), perinatal death, cesarean section, emergency cesarean section, pre-eclampsia, severe preeclampsia, placenta previa, placental abruption, low Apgar score at 5 min, and low pH of umbilical cord artery. We defined SGA as birthweight below 10th percentile for gestational age on the birthweight reference, [29] preterm birth as less than 37 completed weeks of gestation, very preterm birth as less than 32 completed weeks of gestation, and extremely preterm birth as less than 28 completed weeks of gestation [30]. Preeclampsia and severe preeclampsia were diagnosed clinically by obstetricians at each hospital according to the national guideline as systolic/diastolic blood pressure over 140/90 mmHg and 160/110 mmHg that emerges after 20 weeks’ gestation with significant proteinuria (≥300 mg/day), respectively [31]. We defined perinatal death as stillbirth and early neonatal death before day 7 or discharge whichever came first, low Apgar score at 5 min as below 7, and low pH of umbilical cord artery as below 7.1. As a previous study suggested the association between age and birth outcomes may differ by conception method and parity, [19] we considered these as effect modifiers. We categorized conception method into natural and any ART, and parity as primiparous and multiparous.

### Statistical analysis

First, we compared baseline demographics among the four categories of maternal age using test for trend. Next we used Poisson regression to estimate the effect of maternal age on the risk of adverse birth outcomes considering women aged 30–34 as the reference, as well as tested for the trend of the association. Each result was presented as a risk ratio and 95% confidence interval (CI). To confirm our results which were derived based on partially imputed data, we conducted sensitivity analyses restricting the population to only those who had complete data (*n* = 183,084).

Next, using this subset of women with complete data, we examined potential effect modification by parity (primiparous or multiparous) and conception method (conception by ART, conception without ART) on the association between maternal age and risk of adverse birth outcomes. We tested for interaction by including two-way multiplicative interaction terms into the Poisson regression model. Subsequently, Poisson regression analyses were performed stratified by parity and by conception method.

Analyses were adjusted for the following: pre-pregnancy BMI, maternal height, gestational weight gain, conception method, maternal smoking during pregnancy, parity, preexisting hypertension, and abnormal glucose tolerance according to known risk factors for various adverse outcomes based on previous studies [19, 32, 33]. The stratified analysis did not include conception method when stratifying by conception method, or parity when stratifying by parity.

All descriptive and statistical analyses were performed using STATA version 13 (STATA Corp, College Station, TX). Statistical significance was set under 0.05 (including test for interaction), and all statistical tests were two-tailed. The protocol for this study was approved by the Institutional Review Board of the National Center for Child Health and Development on Apr. 18, 2017 (No 1448).

## Results

Maternal demographics and birth outcomes are shown in Table 1. Cesarean-section was the mode of delivery for 117,155 women (32.1%), among whom 52,401 (14.3%) had emergency cesarean section. As for maternal complications, preeclampsia, placenta previa, preterm birth, SGA and low pH of umbilical artery was experienced by 10,689 (2.9%), 7411 (2.0%), 47,727 (13.1%), 16,414 (4.5%), 5509 (1.5%) women, respectively. Women of older age were heavier, taller, more likely to be primiparous, had higher prevalence of pre-existing hypertension and diabetes, and more likely to have conceived through ART (*p*-values for trend: < 0.001).

**Table 1:** Maternal and infant characteristics by maternal age category among 365,417 Japanese women

Mean (SD) or n (%)	Maternal age			
	30–34 (*n* = 204,181)	35–39 (*n* = 131,515)	40–44 (*n* = 28,797)	≥45 (*n* = 924)
Maternal characteristics				
Pre-pregnancy weight (kg)	52.9 (7.4)	54.0 (7.8)	54.8 (8.0)	55.0 (8.4)
Height (cm)	158.4 (4.5)	158.5 (4.6)	158.4 (4.7)	157.9 (4.5)
Pre-pregnancy BMI (kg/m^2^)	21.1 (2.8)	21.5 (3.0)	21.8 (3.1)	22.1 (3.2)
Weight gain during pregnancy (kg)	9.4 (3.5)	9.0 (3.6)	8.8 (3.7)	8.7 (3.9)
Primipara	99,359 (48.7)	55,296 (42.0)	12,788 (44.4)	448 (48.5)
Preexisting hypertension	1241 (0.6)	1576 (1.2)	545 (2.0)	32 (3.5)
Preexisting diabetes or GDM	5184 (2.5)	5009 (3.8)	1520 (5.3)	79 (8.5)
Assisted conception	4963 (2.4)	8641 (6.6)	3987 (13.8)	201 (21.8)
Smoking during pregnancy	11,299 (5.5)	6866 (5.2)	1549 (5.4)	77 (8.3)
Birth outcomes				
Birthweight (g)	2881 (554)	2877 (568)	2861 (588)	2848 (607)
Gestational age at birth (w)	38.2 (2.5)	38.0 (2.5)	37.9 (2.5)	37.7 (2.5)
Infant sex male	105,286 (51.6)	67,695 (51.5)	14,797 (51.4)	478 (51.7)
Pregnancy complications				
Cesarean section	57,881 (28.3)	46,252 (35.2)	12,516 (43.5)	506 (54.8)
Emergency cesarean section	26,812 (13.1)	19,922 (15.1)	5452 (18.9)	215 (23.3)
Preeclampsia	5304 (2.6)	4192 (3.2)	1140 (4.0)	53 (5.7)
Severe preeclampsia	1832 (0.9)	1446 (1.1)	388 (1.3)	20 (2.2)
Placenta previa	3485 (1.7)	3081 (2.3)	805 (2.8)	40 (4.3)
Placental abruption	1908 (0.9)	1338 (1.0)	286 (1.0)	12 (1.3)
Preterm birth	25,754 (12.6)	17,647 (13.4)	4171 (14.5)	155 (16.8)
Very preterm birth	6111 (3.0)	4178 (3.2)	952 (3.3)	29 (3.1)
Extremely preterm birth	2195 (1.1)	1434 (1.1)	287 (1.0)	8 (0.9)
Low birthweight	36,462 (17.9)	23,793 (18.1)	5563 (19.3)	201 (21.8)
Small for gestational age	9067 (4.4)	5846 (4.4)	1449 (5.0)	52 (5.6)
Low apgar at 5 min	4373 (2.1)	2945 (2.2)	690 (2.4)	22 (2.4)
Low pH of umbilical cord artery	1563 (0.8)	1116 (0.8)	2820 (1.0)	10 (10.8)
Perinatal death	1473 (0.7)	924 (0.7)	224 (0.8)	11 (1.2)

*BMI* Body mass index, *GDM* Gestational diabetes mellitus

The associations between maternal age and birth outcomes are shown in Table 2. Compared with women 30–34 years of age, women aged 45 and older had a statistically significant higher risk of cesarean section [adjusted risk ratio (aRR): 1.70, 95% confidence interval: 1.60–1.80)], emergency cesarean section (aRR: 1.54, 95% CI: 1.37–1.73), preeclampsia (aRR: 1.86, 95% CI: 1.43–2.42), severe preeclampsia (aRR: 2.03, 95% CI: 1.31–3.13), placenta previa (aRR: 2.17, 95% CI: 1.60–2.95), preterm birth (aRR: 1.20, 95% CI: 1.04–1.39) and low birthweight (aRR: 1.17, 95% CI: 1.03–1.33). A significant trend in risk was observed for increasing maternal age categories for all outcomes except for placental abruption, very preterm birth, low Apgar score, and perinatal death. Repeating the analysis on a subset of 183,084 women after excluding those whose data underwent imputation showed results consistent with the main analysis (Table 2).

**Table 2:** Risk of adverse outcomes associated with maternal age categories (versus aged 30–34 years)

Outcome	Maternal age	Main analysis (*n* = 365,417)		Women with complete data (*n* = 183,084)
		Crude RR	Multivariate RR^a^	Multivariate RR^a^
Cesarean section	35–39	1.24 (1.23–1.25)	1.20 (1.18–1.21)	1.18 (1.17–1.20)
	40–44	1.53 (1.51–1.56)	1.42 (1.39–1.44)	1.40 (1.37–1.43)
	45–49	1.93 (1.82–2.05)	1.70 (1.60–1.80)	1.53 (1.40–1.70)
	*p for trend*	<0.001	<0.001	<0.001
Emergency cesarean section	35–39	1.15 (1.13–1.17)	1.16 (1.14–1.18)	1.17 (1.14–1.19)
	40–44	1.44 (1.40–1.48)	1.37 (1.33–1.40)	1.38 (1.33–1.43)
	45–49	1.77 (1.58–1.99)	1.54 (1.37–1.73)	1.60 (1.35–1.90)
	*p for trend*	<0.001	<0.001	<0.001
Preeclampsia	35–39	1.23 (1.18–1.28)	1.23 (1.19–1.29)	1.26 (1.19–1.34)
	40–44	1.52 (1.43–1.62)	1.44 (1.35–1.53)	1.48 (1.35–1.63)
	45–49	2.21 (1.70–2.87)	1.86 (1.43–2.42)	2.26 (1.57–3.25)
	*p for trend*	<0.001	<0.001	<0.001
Severe-preeclampsia	35–39	1.22 (1.14–1.31)	1.24 (1.15–1.33)	1.26 (1.14–1.39)
	40–44	1.50 (1.35–1.67)	1.42 (1.27–1.59)	1.45 (1.24–1.70)
	45–49	2.41 (1.56–3.73)	2.03 (1.31–3.13)	1.91 (1.00–3.67)
	*p for trend*	<0.001	<0.001	<0.001
Placenta previa	35–39	1.37 (1.31–1.44)	1.30 (1.24–1.37)	1.20 (1.11–1.30)
	40–44	1.64 (1.52–1.77)	1.46 (1.35–1.58)	1.38 (1.22–1.55)
	45–49	2.54 (1.87–3.44)	2.17 (1.60–2.95)	1.92 (1.16–3.17)
	*p for trend*	<0.001	<0.001	<0.001
Abruption	35–39	1.09 (1.02–1.17)	1.06 (0.99–1.14)	1.11 (0.99–1.25)
	40–44	1.06 (0.94–1.20)	1.02 (0.90–1.16)	1.12 (0.92–1.38)
	45–49	1.39 (0.79–2.44)	1.31 (0.75–2.31)	1.24 (0.47–3.29)
	*p for trend*	0.027	0.179	0.068
Preterm birth (<37w)	35–39	1.06 (1.04–1.08)	1.03 (1.01–1.05)	1.04 (1.01–1.07)
	40–44	1.15 (1.11–1.18)	1.08 (1.05–1.11)	1.09 (1.04–1.14)
	45–49	1.33 (1.15–1.54)	1.20 (1.04–1.39)	1.12 (0.89–1.40)
	*p for trend*	<0.001	<0.001	<0.001
Very preterm birth (<32w)	35–39	1.06 (1.02–1.10)	1.02 (0.98–1.07)	1.03 (0.96–1.10)
	40–44	1.10 (1.03–1.18)	1.03 (0.96–1.11)	1.04 (0.93–1.15)
	45–49	1.05 (0.73–1.50)	0.94 (0.66–1.33)	0.80 (0.43–1.49)
	*p for trend*	<0.001	0.263	0.449
Extremely preterm birth (<28w)	35–39	1.01 (0.95–1.08)	0.96 (0.89–1.04)	0.97 (0.87–1.09)
	40–44	0.93 (0.82–1.05)	0.95 (0.75–0.96)	0.87 (0.72–1.05)
	45–49	0.81 (0.40–1.61)	0.70 (0.35–1.40)	0.23 (0.03–1.60)
	*p for trend*	0.476	0.017	0.139
Low birthweight	35–39	1.01 (1.00–1.03)	1.02 (1.00–1.04)	1.03 (1.01–1.06)
	40–44	1.08 (1.05–1.11)	1.08 (1.05–1.11)	1.09 (1.05–1.14)
	45–49	1.22 (1.08–1.39)	1.17 (1.03–1.33)	1.19 (0.99–1.44)
	*p for trend*	<0.001	<0.001	<0.001
Small for gestational age	35–39	1.00 (0.97–1.03)	1.01 (0.98–1.04)	1.02 (0.98–1.07)
	40–44	1.13 (1.07–1.20)	1.13 (1.07–1.19)	1.08 (1.00–1.17)
	45–49	1.27 (0.97–1.65)	1.20 (0.92–1.57)	1.31 (0.90–1.91)
	*p for trend*	0.001	0.001	0.035
Low Apgar (5 min, <7)	35–39	1.05 (1.00–1.09)	1.02 (0.97–1.07)	1.03 (0.95–1.10)
	40–44	1.12 (1.03–1.21)	1.06 (0.98–1.15)	1.09 (0.97–1.24)
	45–49	1.11 (0.73–1.68)	1.03 (0.68–1.56)	0.59 (0.25–1.40)
	*p for trend*	0.002	0.169	0.255
Low pH of umbilical cord artery (<7.1)	35–39	1.11 (1.03–1.20)	1.13 (1.05–1.22)	1.11 (1.00–1.23)
	40–44	1.28 (1.13–1.45)	1.28 (1.13–1.46)	1.25 (1.06–1.48)
	45–49	1.41 (0.76–2.62)	1.35 (0.73–2.51)	1.18 (0.49–2.84)
	*p for trend*	<0.001	<0.001	0.003
Perinatal death	35–39	0.97 (0.90–1.06)	0.98 (0.90–1.07)	1.01 (0.89–1.15)
	40–44	1.08 (0.94–1.24)	1.10 (0.96–1.27)	1.06 (0.85–1.32)
	45–49	1.65 (0.92–2.98)	1.70 (0.94–3.07)	1.51 (0.57–4.02)
	*p for trend*	0.498	0.324	0.555

^a^Adjusted by pre-pregnancy BMI, maternal height, gestational weight gain, conception method, maternal smoking during pregnancy, parity, preexisting hypertension, and preexisting diabetes or GDM

The associations between maternal age and birth outcomes stratified dichotomously by maternal parity and conception method are shown in Table 3. The effect of advanced age on increased risk of cesarean section and emergency cesarean section were significantly greater among primiparous women than among multiparous women. Conversely, the effect of age on increased risk of preeclampsia, and severe preeclampsia were significant greater among multiparous women. Evidence for a heterogeneous effect of advanced age on the risk of preterm birth, very preterm birth, extremely preterm birth, low birthweight, placental abruption, placental previa, low Apgar score, low pH of umbilical cord artery and perinatal death between primiparous and multiparous women was not observed.

**Table 3:** Risk of adverse outcomes associated with advanced maternal age (compared with women aged 30–34 years) stratified analysis by conception method and parity

Outcome	Maternal age (years)	ART^a^			Parity^b^		
		Yes (*n* = 9289)	No (*n* = 170,495)	p for interaction	Primiparous (*n* = 84,196)	Multiparous (*n* = 98,888)	p for interaction
Cesarean section	35–39	1.19 (1.12–1.27)	1.18 (1.17–1.20)		1.30 (1.25–1.31)	1.12 (1.10–1.14)	
	40–44	1.49 (1.40–1.59)	1.38 (1.35–1.41)		1.66 (1.60–1.70)	1.22 (1.19–1.26)	
	45–49	1.71 (1.45–2.01)	1.49 (1.33–1.67)		2.13 (1.92–2.37)	1.10 (0.93–1.30)	
	*p for trend*	<0.001	<0.001	0.183	<0.001	<0.001	<0.001
Emergency cesarean section	35–39	1.15 (1.04–1.27)	1.16 (1.13–1.19)		1.21 (1.17–1.24)	1.11 (1.07–1.15)	
	40–44	1.20 (1.07–1.34)	1.41 (1.35–1.47)		1.46 (1.40–1.54)	1.28 (1.20–1.36)	
	45–49	1.25 (0.87–1.80)	1.71 (1.41–2.08)		1.74 (1.41–2.14)	1.39 (1.03–1.89)	
	*p for trend*	0.001	<0.001	0.017	<0.001	<0.001	<0.001
Preeclampsia	35–39	1.11 (0.87–1.43)	1.27 (1.19–1.34)		1.20 (1.11–1.30)	1.37 (1.25–1.50)	
	40–44	1.12 (0.84–1.50)	1.53 (1.39–1.69)		1.24 (1.09–1.42)	1.82 (1.60–2.08)	
	45–49	1.57 (0.71–3.47)	2.48 (1.66–3.70)		1.33 (0.72–2.45)	3.62 (2.34–5.61)	
	*p for trend*	0.294	<0.001	0.03	<0.001	<0.001	<0.001
Severe-preeclampsia	35–39	1.19 (0.80–1.77)	1.26 (1.14–1.39)		1.23 (1.08–1.39)	1.33 (1.13–1.55)	
	40–44	1.13 (0.71–1.80)	1.51 (1.28–1.79)		1.20 (0.96–1.49)	1.84 (1.46–2.32)	
	45–49	2.82 (1.05–7.58)	1.51 (0.63–3.63)		1.69 (0.71–3.99)	2.31 (0.86–6.21)	
	*p for trend*	0.293	<0.001	0.381	0.002	<0.001	0.006
Placenta previa	35–39	1.07 (0.85–1.35)	1.25 (1.16–1.34)		1.33 (1.20–1.48)	1.17 (1.07–1.28)	
	40–44	1.10 (0.84–1.45)	1.44 (1.27–1.62)		1.45 (1.22–1.71)	1.33 (1.15–1.55)	
	45–49	1.11 (0.42–2.94)	2.60 (1.59–4.27)		2.31 (1.25–4.28)	1.98 (1.04–3.75)	
	*p for trend*	0.487	<0.001	0.021	<0.001	<0.001	0.152
Placental abruption	35–39	2.08 (1.20–3.59)	1.09 (0.97–1.21)		1.26 (1.07–1.47)	1.04 (0.90–1.19)	
	40–44	0.85 (0.39–1.84)	1.18 (0.98–1.43)		1.05 (0.78–1.42)	1.14 (0.90–1.44)	
	45–49	1.79 (0.24–13.2)	1.69 (0.71–4.03)		1.36 (0.34–5.48)	1.76 (0.67–4.64)	
	*p for trend*	0.989	0.027	0.594	0.054	0.231	0.753
Preterm birth (<37w)	35–39	0.90 (0.81–1.12)	1.04 (1.01–1.07)		1.03 (0.99–1.07)	1.04 (1.01–1.08)	
	40–44	0.76 (0.65–0.88)	1.13 (1.08–1.18)		1.04 (0.97–1.12)	1.12 (1.06–1.19)	
	45–49	0.87 (0.52–1.44)	1.16 (0.90–1.50)		1.25 (0.90–1.73)	1.01 (0.74–1.40)	
	*p for trend*	0.001	<0.001	<0.001	0.104	<0.001	0.252
Very preterm birth (<32w)	35–39	0.82 (0.64–1.06)	1.03 (0.97–1.11)		1.01 (0.92–1.12)	1.03 (0.95–1.13)	
	40–44	0.60 (0.42–0.86)	1.10 (0.99–1.23)		0.98 (0.83–1.16)	1.08 (0.95–1.24)	
	45–49	0.57 (0.14–2.25)	0.84 (0.42–1.68)		1.24 (0.59–2.62)	0.44 (0.14–1.34)	
	*p for trend*	0.004	0.108	0.001	0.945	0.357	0.791
Extremely preterm birth (<28w)	35–39	0.66 (0.43–1.00)	0.99 (0.88–1.12)		0.97 (0.82–1.15)	0.97 (0.83–1.13)	
	40–44	0.48 (0.26–0.87)	0.93 (0.76–1.23)		0.76 (0.56–1.03)	0.97 (0.76–1.23)	
	45–49	–	0.31 (0.04–2.17)		0.47 (0.07–3.32)	–	
	*p for trend*	0.005	0.452	0.013	0.120	0.513	0.758
Low birthweight	35–39	0.99 (0.89–1.10)	1.03 (1.01–1.06)		1.04 (1.01–1.08)	1.02 (0.99–1.06)	
	40–44	0.89 (0.78–1.01)	1.12 (1.08–1.17)		1.04 (0.98–1.10)	1.14 (1.08–1.20)	
	45–49	0.98 (0.63–1.51)	1.24 (1.01–1.52)		1.15 (0.87–1.51)	1.23 (0.96–1.58)	
	*p for trend*	0.096	<0.001	0.004	0.011	<0.001	0.161
Small for gestational age	35–39	0.85 (0.68–1.06)	1.03 (0.98–1.08)		1.00 (0.93–1.07)	1.04 (0.98–1.10)	
	40–44	0.72 (0.54–0.96)	1.12 (1.03–1.22)		0.94 (0.83–1.07)	1.20 (1.08–1.32)	
	45–49	0.66 (0.22–2.01)	1.44 (0.97–2.13)		1.02 (0.54–1.93)	1.54 (0.97–2.44)	
	*p for trend*	0.019	0.005	0.003	0.546	0.001	0.017
Low Apgar (5 min, <7)	35–39	0.85 (0.62–1.17)	1.03 (0.96–1.11)		1.06 (0.95–1.17)	1.00 (0.91–1.10)	
	40–44	0.71 (0.48–1.07)	1.14 (1.01–1.30)		0.99 (0.82–1.20)	1.18 (1.01–1.39)	
	45–49	0.43 (0.06–3.03)	0.61 (0.23–1.60)		0.24 (0.03–1.71)	0.90 (0.34–2.37)	
	*p for trend*	0.069	0.092	0.041	0.835	0.179	0.66
Low pH of umbilical cord artery (<7.1)	35–39	0.94 (0.58–1.51)	1.12 (1.01–1.24)		1.03 (0.89–1.19)	1.20 (1.04–1.39)	
	40–44	1.23 (0.72–2.10)	1.24 (1.04–1.49)		1.20 (0.95–1.52)	1.33 (1.04–1.68)	
	45–49	–	1.52 (0.64–3.64)		1.34 (0.44–4.14)	1.02 (0.25–4.09)	
	*p for trend*	0.689	0.003	0.756	0.185	0.003	0.138
Perinatal death	35–39	0.71 (0.38–1.31)	1.02 (0.90–1.16)		0.97 (0.80–1.17)	1.04 (0.88–1.23)	
	40–44	0.60 (0.27–1.34)	1.11 (0.88–1.39)		1.08 (0.78–1.50)	1.05 (0.78–1.41)	
	45–49	–	1.87 (0.71–4.93)		0.83 (0.12–5.86)	2.11 (0.69–6.49)	
	*p for trend*	0.137	0.326	0.102	0.910	0.485	0.853

^a^Adjusted by pre-pregnancy BMI, maternal height, gestational weight gain, maternal smoking during pregnancy, parity, preexisting hypertension, and preexisting diabetes or GDM

^b^Adjusted by pre-pregnancy BMI, maternal height, gestational weight gain, conception method, maternal smoking during pregnancy, preexisting hypertension, and preexisting diabetes or GDM

The effect of advanced age on increased risk of emergency cesarean section, preeclampsia, placental previa, preterm birth, very preterm birth, extremely preterm birth, low birthweight, and low Apgar score were significantly greater among women who conceived without ART than among those who conceived with ART. Women who conceived by ART did not show stronger effects of advanced age on risk of adverse outcomes than among women who conceived without ART.

## Discussion

Using a large nation-wide obstetrics database, we showed pregnant women aged 45 years and older had a 1.5–2 fold greater risk of experiencing maternal morbidities compared to younger women (age 30–34), including risk of cesarean section, preeclampsia, severe preeclampsia, and placenta previa. The risk of neonatal outcomes such as preterm birth, low birthweight, SGA and low pH of umbilical cord artery were relatively smaller (3–20%) or even null. Furthermore, we found that the effect of advanced age differed by conception method and parity. The effect of age on increased pregnancy/birth outcome risks were generally smaller among women who conceived with ART than those without ART. Regarding parity, the effect of age on the risk of cesarean section and emergency cesarean section were significantly greater among primiparous women, while its effect on preeclampsia risk was significantly greater among multiparous women.

Consistent with several previous studies, [8, 9, 12, 13, 19] our study showed positive association between risk of preterm delivery and maternal age. Although we demonstrated the estimated risk to be largest for those women aged 45 and older, the difference in risk compared to women aged 30–34 was still relatively small. Interestingly, we found that this effect of age differed by conception method. That is, while the risk increased with age in women who conceived without ART, it appeared to decrease in women who conceived with ART. These findings were similar to an Australian study which showed increased maternal age was associated with increased risk of preterm birth only in women who conceived without ART [19]. As for very preterm and extremely preterm births specifically, similar effect modification by ART were observed, but showed less precision due to the smaller numbers of these outcomes. These finding may suggest, that while there is a positive association between maternal age and risk of preterm birth, younger women who conceived through ART may have higher risk of preterm birth compared to those who conceive through ART at older ages.

A similar pattern of effect modification by conception method was observed for risk of placenta previa. The effect of maternal age was stronger among those who conceived without ART. As the proportion of women conceiving with ART also increases with age, these results may also reflect the increased clinical risk of adverse birth outcomes among young women who needed ART to conceive.

In our study older maternal age was significantly associated with risk of cesarean section, including emergency cesarean section, where women aged 45 and older had the highest risk, consistent with findings from previous studies [9, 13, 19, 32, 33]. We found that the effects of increased age were significantly greater among primiparous women than multiparous women, consistent with two previous studies [19, 33]. This effect modification may be due to higher prevalence of elective cesarean section by request among primiparous women of advanced age [32, 34]. It could also be due to primiparous women having a greater increase in risk of prolonged labor or non-reassuring fetal status requiring emergency cesarean section with increasing age. However as the JSOG-DB lacked detailed information on indication for cesarean section, we could not verify this hypothesis in our study.

The risk of preeclampsia, including severe preeclampsia, was also increased among women of advanced age, especially among those 45 years and over in our study. The effect of maternal age on preeclampsia and severe preeclampsia were greater among multiparous women than primiparous women, which is in contrast to a previous study of 1404 US women which reported a similar effect of age on risk of preeclampsia in both primiparous and multiparous women [35]. One possible explanation for the smaller effect of age observed in primiparous women in our study is the recent use of low dose aspirin in women with high risk of preeclampsia, [36] which was likely more common in the current study than the US study conducted 20 years ago. As both primiparity and advanced age are considered strong risk factors for preeclampsia, [37] primiparous women of advanced age may be more likely to receive such medication compared to multiparous women. If this is the case, our study suggests that such practice should be considered for not only primiparous women, but also for multiparous women of advanced age.

Our success in assembling high quality data on a large cohort of pregnant women and births led to many strengths, including the ability to assess rare outcomes such as severe preeclampsia or placental abruption, as well as conduct detailed analyses stratified by method of conception and parity.

Nonetheless, we acknowledge several limitations of our study. First, data on conception method were from records at the delivery hospitals, which in some cases could have been based on self-report from the mother, leading to underreporting of ART usage. Although previous studies demonstrated high positive predictive value of self-reported conception method on actual method, we cannot exclude the possibility of misclassification bias [38, 39]. Second, because our database was based on tertiary hospitals, our study population likely comprised a higher proportion of high risk pregnancies leading to potential underestimation of the effect of advanced age on adverse outcomes compared to the general population. To reduce this bias, we excluded women with higher risk such as multiple pregnancy, fetal anomaly; maternal characteristics associated with advanced age pregnancies and risk of adverse pregnancy outcomes were adjusted for in the multivariate analysis, such as preexisting hypertension and abnormal glucose tolerance. Furthermore, we confirmed that our results did not change after adjusting for institution (data not shown). However, further population-based studies should be performed for replication and to clarify the generalizability of our findings. Third, while oocyte donation is one method for conception more popular among women of very advanced age, [40] and women who conceived by oocyte donation are reported to have higher risk of adverse birth outcomes, [41] our database did not include information on the type of ART. As ART is becoming more popular, and the choice of ART method is becoming more complex, future studies using more detailed information of oocyte donation and ART are needed. Finally, our analyses were unable to take into account social economic status (SES), as our database did not collect relevant information. As pregnant women of advanced age would be more likely to be multiparous and conceive without ART if of lower SES, it is possible SES would have biased our findings. Future studies that have adequate measurements of SES should be conducted to check whether our findings can be replicated.

## Conclusions

In conclusion, women of advanced age, especially those aged 45 years and older have an elevated risk of adverse outcomes such as cesarean section, preeclampsia, placenta previa, preterm birth, and low birthweight. However, the magnitude of association between age and adverse outcomes differed by parity and conception method. Such findings should be taken into account when conducting antenatal counseling in clinical settings for women with very advanced women.

## Abbreviations

ART: Artificial reproductive technologies
BMI: Body mass index
JSOG-DB: Japan Society of Obstetrics and Gynecology Perinatal Database
SES: Social economic status
SGA: Small for gestational age
